# Salt taste perception, dietary salt intake, cardiovascular health and genetic variation in Zambian adults with HIV

**DOI:** 10.3389/fphys.2025.1616785

**Published:** 2025-10-14

**Authors:** Sepiso K. Masenga, Catherine A.-M. Graham, Jonathan Nixon, Joreen P. Povia, Annet Kirabo, Leta Pilic

**Affiliations:** ^1^ Department of Physiological Sciences, Mulungushi University School of Medicine and Health Sciences, Kabwe, Zambia; ^2^ Precision Nutrition, Lake Lucerne Institute (LLUI), Lucerne, Switzerland; ^3^ St Mary’s University, London, United Kingdom; ^4^ Department of Molecular Physiology and Biophysics, Vanderbilt University, Nashville, TN, United States; ^5^ Department of Medicine, Vanderbilt Center for Immunobiology, Vanderbilt Institute for Infection, Immunology and Inflammation, Vanderbilt Institute for Global Health, Vanderbilt University Medical Center, Nashville, TN, United States; ^6^ Optimyse Nutrition Ltd., Radlett, United Kingdom

**Keywords:** SCNN1B rs239345, TRPV1 rs4790522, HIV, dietary salt, blood pressure, Zambia, cardiovascular health

## Abstract

**Introduction:**

Cardiovascular diseases (CVDs) are the leading cause of noncommunicable mortality in sub-Saharan Africa (SSA), driven in part by excessive salt intake. People living with HIV (PLWH) face heightened CVD risks due to hypertension and potential taste dysfunction, yet genetic and sensory drivers of salt intake in this population remain understudied. This study primarily explores differences between salt taste perception and dietary salt intake. The secondary aim was to determine if genetic variation influences the above and health markers of CVD.

**Methods:**

Seventy-nine Zambian adults (37 PLWH, 42 healthy controls (HC)) underwent salt taste threshold/preference assessments, 24-h dietary recalls, and genotyping. Salt intensity/pleasantness was rated using visual analogue scales, and area-under-the-curve (AUC) values were calculated. Systolic and diastolic blood pressure, pulse rate, and body mass index (BMI) were measured.

**Results:**

PLWH consumed more salt than HC (median: 9.01 vs. 7.11 g/day, p = 0.041) and exhibited reduced salt intensity perception (AUC: 94.8 vs. 109.2, p = 0.02). Genetic analyses revealed that PLWH with the TRPV1 rs4790522 AC/CC genotype consumed more salt (9.4 vs. 7.3 g/day, p < 0.05) and those with the AA genotype had lower BMIs than HC (21.0 vs. 27.9 kg/m^2^, p = 0.001). The SCNN1B rs239345 AT/AA genotype in PLWH was linked to elevated systolic/diastolic blood pressure (142.0/92.0 mmHg) at high salt intake. No direct correlations emerged between taste thresholds and salt intake (p > 0.05).

**Conclusion:**

PLWH consumed more salt than HC, which may be driven by a reduced salt perception. Additionally, differences were found in the association between the TRPV1 rs4790522 SNP, salt intake and BMI profiles, between PLWH and HC, suggesting a gene-environment-disease interaction. Future research should validate these associations in larger cohorts and explore interventions addressing genetic and sensory contributors to hypertension in high-risk SSA populations.

## 1 Introduction

Cardiovascular diseases (CVD) are the leading cause of death from noncommunicable diseases (NCDs) in sub-Saharan Africa (SSA). It is estimated that the region accounts for approximately 80% of deaths from CVDs globally (Shumba et al., 2024). The number one risk factor for CVD is a high intake of sodium or salt due to its causal relationship with elevated blood pressure (DiNicolantonio et al., 2017). Salt is the leading dietary risk factor contributing to global morbidity and mortality. It was estimated that the mean global sodium consumption in 2019 was 4,310 mg/day (10.78 g/day salt), exceeding the WHO recommended intake of 2000 mg/day (Afshin et al., 2019). In Africa, the estimated sodium intake in 2019 was 2,687 mg/day (6.72 g/day salt) (WHO, 2023), within SSA salt intake ranges from 6.8 to 11.3 g/day (Oyebode et al., 2016). The influencers of salt intake in a SSA cohort are not comprehensively understood, this coupled with the high CVD death rate emphasises the requirement for further research.

Salt taste perception and preference are drivers of dietary salt intake (Tan et al., 2021). More specifically, impaired salt taste perception may lead to an increase in dietary salt intake, as individuals may use more salt to achieve the desired taste (Azinge et al., 2011). This impairment can be a consequence of various factors, including body mass index (BMI), disease status, medication, and genetic variation (Fasunla et al., 2016; Pilic and Mavrommatis, 2018; Bigiani, 2020). An increased BMI has been associated with a decrease in global taste sensitivity (Vignini et al., 2019), however research is scarce in a SSA cohort with no literature on people living with human immunodeficiency virus (HIV, PLWH).

HIV is an epidemic, with the greatest prevalence in SSA (Moyo et al., 2023). Although research is scarce, PLWH have been reported to have an impaired taste ability (Graham et al., 1995; Fasunla et al., 2017), potentially due to HIV’s affinity for disrupting nervous tissues, an increased susceptibility to upper aerodigestive tract infections, or by the use of antiretroviral therapy (ART; Fasunla et al., 2016; Verma et al., 2017). In PLWH salt taste ability is of great importance due to the association between HIV and hypertension (Masenga et al., 2019; Pangmekeh et al., 2019) and separately salt intake and hypertension (Grillo et al., 2019). If salt taste perception is impaired in PLWH, then a potentially higher salt intake could worsen health outcomes in a population already at increased risk of hypertension and CVD.

Salt taste perception is partially determined by genetic variations in salt taste receptors. Two membrane receptors have been identified: the epithelial sodium channel (ENaC; encoded by products of the Sodium Channel Epithelial 1 Subunit *(SCNN*1*A, SCNN*1*B, SCNN*1*G*, and *SCNN*1*D* genes) and the transient receptor potential cation channel subfamily V member 1 (TRPV1, encoded by the *TRPV1* gene) (Tapanee et al., 2021). Salt taste is transduced by a sodium-specific (amiloride (Am)-sensitive) pathway and a non-selective (Am-insensitive) pathway (Rhyu et al., 2024). Single nucleotide polymorphisms (SNPs), namely, rs239345 *SNN*1*B,* related to the Am-sensitive pathway, and rs8065080 *TRPV*1, related to the Am-insensitive pathway, have been correlated with salt taste perception in various cohorts, including Canadian, Iranian, Spanish, United States, and United Kingdom adults (Dias et al., 2013; Barragán et al., 2018; Chamoun et al., 2018; Pilic et al., 2020; Mohammadifard et al., 2023). However, despite the important implications of these findings, research focusing on the genetic basis of salt taste perception and its impact on dietary salt intake remains limited in SSA, despite the frequency of the minor allele in SSA populations being high. This coupled with the heightened CVD risk, particularly within PLWH, makes it imperative that further research is conducted.

Therefore, the primary aim of this study was to explore and compare, in a Zambian population of healthy adults (controls; HC) and PLWH, differences between salt taste perception and dietary salt intake. The secondary aim was to determine if genetic variation influences salt perception and intake and health markers of CVD.

## 2 Methods

### 2.1 Study design and participants

This was a pilot study involving Zambian adults, 37 PLWH and 42 HC matched for sex and BMI Participants were recruited through advertisements and during in-hospital visits.

### 2.2 Eligibility and ethics

Participants were excluded with a history of any chronic disease diagnosis apart from HIV (as listed in the International Statistical Classification of Diseases and Related Health Problems 10th Revision (ICD-10, 2019) or the use of any medications besides ART. All PLWH were on ART form more than 1 year and virally suppressed. In addition, pregnant and lactating women, and participants who have suffered a previous illness or incident that permanently alters taste, for example, stroke, head trauma, lasting effects from COVID-19, were also excluded from the study. All procedures involving human participants were approved by the Mulungushi University Research Ethics Committee (reference number: SMHS-MU1-2022-10). Written informed consent was obtained from each participant before the data collection.

During testing, all participants completed the salt taste threshold and preference assessment, provided a saliva sample for genotyping, and completed a 24-h dietary recall providing information on salt eating habits.

### 2.3 Demographic data

Demographic data (age (years), sex (m/f/prefer not to say), and ethnicity) together with smoking habits and health status information were collected using a questionnaire.

### 2.4 Anthropometric measurements

Height (m) and weight (kg) were measured using a stadiometer and body mass index (BMI) were calculated using the equation kg/m^2^.

### 2.5 Blood pressure and pulse rate

Blood pressure (mmHg) and pulse rate was recorded using a certified automatic monitor (Omron HEM-7120; Omron Healthcare Co., Ltd., Kyoto, Japan) while participants sat upright, ensuring their backs rested against the chair and their feet were flat on the ground, with arms aligned to the level of the heart.

### 2.6 Taste threshold

Six concentrations of sodium chloride (0.0, 5.0, 15.0, 30.0, 60.0, 120.0 and 240.0 g/L) diluted with spring water were prepared on the day of testing. The solutions were presented to participants in increasing concentrations. Participants rinsed their mouth with water before tasting each solution and then swirled the test solutions in the mouth for approximately 10 s before exporating it. The recognition threshold, the concentration at which the respondent could distinguish the salty taste from water, and the preferred sample, the concentration the respondents chose as their favorite, were recorded, as per (Eriksson et al., 2019; Tapanee et al., 2021).

### 2.7 Taste preference and self-reported eating habit

Tomato soup was prepared by mixing spring water with tomato passata (Pastificio Riscossa, Corato, Italy; www.riscossa.it) in 1:1 ratio. Salt (NaCl) was added to manipulate the final salt concentrations of soup: 0.15%, 0.30%, 0.50%, 1%, 1.5% and 2% (w/w). The soups were presented to participants in increasing concentrations. Participants tasted each soup and rinsed their mouth with water between each sample. Saltiness and pleasantness of each of the soups was rated on a 100 mm visual analogue scale (VAS) ranging from “not at all salty” (0%) to extremely salty (100%) and “very unpleasant” (0%) to “very pleasant” (100%).

### 2.8 Single nucleotide polymorphism (SNP) genotyping

Genotyping was performed according to Pilic and Mavrommatis, (2018). Pre-designed TaqMan^®^ SNP genotyping assays for: *SCNN1B* rs239345, *TRPV1* rs8065080, rs4790522 and the StepOnePlus thermocycler (Applied Biosystems, CA, United States) were used. The primers and the probes were pre-designed by Applied Biosystems with the following codes (C___2387896_30, C__11679656_10, C___3269499_10). Call rates were higher than 95% and all SNPs were in Hardy Weinberg equilibrium (p > 0.05). *SCNN1B* rs239345 minor allele frequency (MAF) (A) was 42%, the *TRPV1* rs8065080 (C) 8% and rs4790522 (C) 49%. rs8065080 was excluded from further analysis considering low MAF. SNPs were selected based on either a high know MAF in an African population or previous association with both salt taste and blood pressure in multicultural populations (Dias et al., 2013; Barragán et al., 2018; Chamoun et al., 2018; Pilic et al., 2020; Mohammadifard et al., 2023).

### 2.9 Dietary salt intake

Dietary salt intake was assessed with a 24-h dietary recall, based on the United States Department of Agriculture (USDA) 5-step multiple pass method and administered via an online platform (Jisc Online Survey; (Rhodes et al., 2013). The method includes a forgotten food list, and in addition to the typically forgotten foods such as tea, coffee, non-alcoholic and alcoholic beverages, sweets and snacks, high salt foodstuffs were also included, such as pickled vegetables, deli meats, smoked fish, cheese, bread and condiments. Participants were also asked to provide information about the quantity of the stock cubes or gravy granules, if used, while cooking. Discretionary salt use was assessed asking questions on adding salt while cooking and at the table, with participants providing the quantities of added salt. Participants were asked to provide food portion size by fist size (Gibson et al., 2016; Amoutzopoulos et al., 2020). Energy and nutrient intake were calculated using a nutritional analysis software (Nutritics, Nutritics LTD, Dublin, Ireland). Total sodium intake (non-discretionary and discretionary) was calculated as an average of sodium intake, expressed as both absolute and energy adjusted (mg sodium per 1,000 kcal).

### 2.10 Statistical analysis

Continuous variables are presented as mean ± standard deviation (SD) or median ± interquartile range (IQR) and were tested for normality with Shapiro-Wilk test. Categorical variables are presented as absolute (relative) frequencies, grouped by median value.

Differences in baseline characteristics by health status (PLWH vs. HC) were assessed using an independent-samples t-test (with Levene’s test for equality of variance), Mann Whitney U test or Fisher’s exact test, as appropriate. Individual ratings of saltiness (intensity) and pleasantness in soup (mm) were plotted and the area under the curve (AUC) calculated using GraphPad Prism (Version 10.4.1; GraphPad Software Inc.). Differences in AUC values were tested using the Independent T-test or Mann Whitney U as appropriate. AUC values for intensity and pleasantness, and intensity and pleasantness against salt intake (g/day) were plotted using Pearson’s correlation to assess correlations within PLWH and HC.

To assess thresholds and preferred concentration sample, considering there is no universal cut-off point to distinguish between the participants with low and high salt taste thresholds, the median was used as cut-offs. Participants with detection threshold <0.467 g/L and a preferred sample concentration of <3.74 g/L were considered to have low thresholds. Furthermore, to explore the effects of disease, two-way ANOVAs were used to test threshold and preferred concentration with AUC intensity and pleasantness by disease state.

To assess health variables, BMI, SBP, DBP, and pulse were assessed by disease state using the Independent T-Test or Mann Whitney U test as appropriate. Then, dietary salt intake was dichotomized by median (<8/≥8 g/d), and disease state and salt intake were assessed with health variables as outcomes. A two-way ANOVA was used throughout. The same approach was taken with threshold and preferred sample cut-offs.

Furthermore, to assess genetic variation, the following groupings were applied: TRPV1 rs4790522 AA and AC/CC genotypes, SCNN1B rs239345 TT and AT/AA genotypes, as per previous literature findings (Tapanee et al., 2021). Dietary salt intake (<8/≥8 g/d) were assessed by genotype frequency, using the Chi Squared test, and then the interaction of disease status was assessed by two-way ANOVA including genotype and disease state with dietary salt intake (g/d) as the outcome variable. The same approach was taken with threshold and preferred sample data.

Lastly, health variables, BMI, SBP, DBP, and pulse, were assessed by genotype grouping. Then, as exploratory and hypothesis generating analysis only, the impact of genotype and salt intake was assessed by health status with BMI, SBP, DBP, and pulse as outcome variables. Two-way ANOVA was used throughout.

All data were corrected for multiple testing, with main effect and pairwise comparison results presented throughout. All analysis were performed using GraphPad Prism (Version 10.4.1; GraphPad Software Inc.). All tests were two-tailed, with p < 0.05 considered statistically significant.

## 3 Results

### 3.1 Participant characteristics

A total of 37 PLWH (50% female), aged 46.9 ± 10.4 years, with a BMI of 22.7 ± 3.7 kg/m^2^, and 42 healthy controls (HC; 49% female), aged 31.2 ± 9.9 years, with a BMI of 24.5 ± 4.9 kg/m^2^ participated (Tables 1, 2). All PLWH (91.9%) in exception of three, were on integrase strand transfer inhibitor (INSTI)-based ART. Median (interquartile range (IQR) duration on ART for PLWH was 12 (7–15) years. Mean age differed between groups (p < 0.001; Table 1). There were no differences in sex (Table 1), BMI (Table6), or TRPV1 and SCNN1B genotype distribution between cohorts (p > 0.05; Table 1).

**TABLE 1:** Participant characteristics.

Variable		HC	PLWH	p-value
n (%)		42 (53.2%)	37 (46.8%)	
Age (*years* mean ± SD)		31.2 ± 9.9	46.9 ± 10.4	**<0.001**
Sex, n (%)				
*Male*		21 (52.5)	19 (47.5)	0.905
*Female*		21 (53.8)	18 (46.2)	
ART Regime, *n(%)*				-
*INSTI-based*		*NA*	34 (91.9)	
*PI-based*		*NA*	3 (8.1)	
ART Duration, (*years median, IQR)*		*NA*	12 (7–15)	-
Genotype Frequencies n (%)				
*TRPV1* rs4790522	AA	9 (12.0)	9 (12.0)	0.207
	AC/CC	33 (32.0)	24 (44.0)	
*SCNN1B* rs239345	TT	11 (13.7)	10 (15.1)	0.312
	AT/AA	31 (28.8)	21 (42.5)	

BMI, body mass index; HC; healthy controls, N, participant number; PLWH, people living with HIV; ART, antiretroviral therapy; INSTI, integrase strand transfer inhibitor; PI, protease inhibitor; IQR, interquartile range; SCNN1B, Sodium Channel Epithelial 1 Subunit; SD, standard deviation; TRPV1, transient receptor potential cation channel subfamily V member 1 gene. P-value; significance level <0.05 (values in bold are significant). The Independent T-test, Mann Whitney U Test, or one-way ANOVA, where used to assess group differences.

Bold values are indicated where the p value <0.05.

**TABLE 2:** Salt taste assessments in HC and PLWH.

Variable	HC	PLWH	p-value
Dietary Salt (g/d: Median (IQR))	7.11 (3.88)	9.01 (3.56)	**0.041**
Detection threshold (n, %)			
≤50^TH^ Perc. (≤0.467 g/L)	9,41	13,59	0.618
>50^th^ Perc. (>0.467 g/L)	28,49	29,51	
Preferred sample (n, %)			
≤50^TH^ Perc. (≤3.47 g/L)	9,36	16,64	0.230
>50^th^ Perc. (>3.74 g/L)	28,52	26,48	
AUC Intensity	109.2	94.8	**0.023**
AUC Pleasantness	86.0	80.2	0.436

AUC; area under the curve, HC; healthy control, IQR; interquartile range, n; participant number, Perc; percentile, PLWH; people living with HIV, p-value; significance level < 0.05 (values in bold are significant). The Independent T-test, Mann Whitney U Test, or one-way ANOVA, where used to assess group differences.

Bold values are indicated where the p value <0.05.

### 3.2 Salt intake and salt taste

**FIGURE 1:**
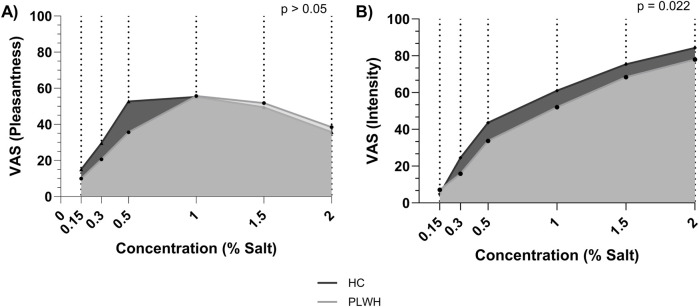
Area under the curve for **(A)** pleasantness and **(B)** intensity of salt threshold by concentration. HC; Healthy Control, PLWH; people living with HIV, VAS; visual analogue scale. P-value; significance level <0.05 (values in bold are significant). The Independent T-test was used to assess group differences.

**FIGURE 2:**
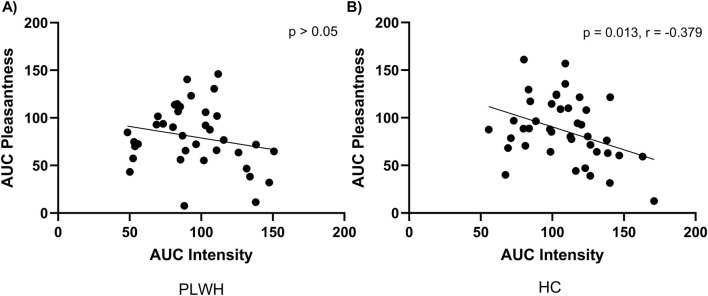
Area under the curve for pleasantness and intensity of salt within **(A)** PLWH and **(B)** HC. AUC; area under the curve, HC; Healthy Control, PLWH; people living with HIV. P-value; significance level <0.05 (values in bold are significant). Pearson’s correlation was used throughout.

Dietary salt intake differed based on disease status, PLWH consumed more salt (9.01 (3.56) g/d) than PLWH (7.11 (3.88) g/L; p = 0.041; Table 2). Salt detection threshold and preferred threshold sample did not differ between groups (p > 0.05). For the intensity of salt perceived, AUC was higher in HC than PLWH (109.2 ± 26.7, 94.8 ± 28.4; p = 0.02; Figure 1). No difference was found regarding the pleasantness of salt (p > 0.05). Intensity and pleasantness were negatively correlated in HC (p = 0.013, r = −0.379), no difference was found in PLWH (p > 0.05; Figure 2).

Total salt intake did not differ based on threshold nor preferred sample within or between cohorts (p > 0.05: Table 3).

**TABLE 3:** Salt intake by threshold and preferred sample.

	PLWH			HC			
	Mean	SD	n	Mean	SD	n	p-value
Threshold							
<50%	7.5	2.1	9	6.6	2.5	13	1.000
>50%	9.1	3.9	28	8.2	3.9	29	
Preferred							
<50%	9.1	3.4	9	6.9	3.1	16	0.317
>50%	8.6	3.7	28	8.2	3.8	26	

HC; healthy control, PLWH; people living with HIV, SD; standard deviation. P-value; significance level <0.05 (values in bold are significant). Two-way ANOVA, was used throughout.

**FIGURE 3:**
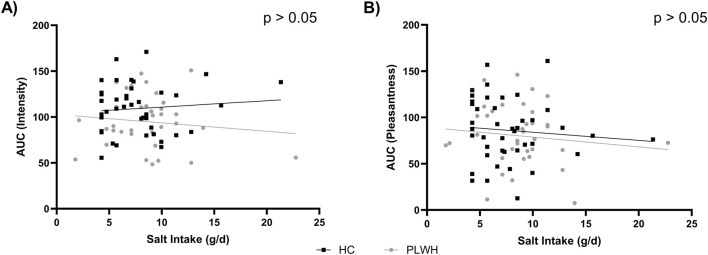
Salt intake by **(A)** intensity AUC and **(B)** pleasantness AUC in PLWH and HC. AUC; area under the curve, HC; Healthy Control, PLWH; people living with HIV. P-value; significance level <0.05 (only significant values shown). Pearson’s correlation was used throughout.

Total salt intake was not correlated with the AUC for intensity or pleasantness (p > 0.05; Figure 3).

Disease status and threshold or preferred sample did not influence intensity of salt perceived (p > 0.05). However, pairwise comparisons revealed HCs with a low threshold (<50%) perceived a higher intensity (122.5 ± 25.6) than PLWH with a high threshold (94.9 ± 30.3; >50%; p = 0.016), and HCs with a low concentration of preferred sample (<50%) perceived a higher intensity (125.6 ± 17.0) than HC (99.2 ± 26.8) and PLWH (91.7 ± 27.5) with a high concentration (>50%) preferred sample (p = <0.001, 0.011 respectively; Table 4).

**TABLE 4:** Pleasantness and intensity by threshold and preferred sample concentration.

	Intensity	PLWH			HC			p-value
		AUC	SD	n	AUC	SD	n	
Threshold	<50%	94.4	22.9	9	*122.5	25.6	13	0.156
	>50%	*94.9	30.3	28	103.3	25.4	29	
Preferred	<50%	104.5	30.3	9	*^+^125.6	17.0	16	0.294
	>50%	^+^91.7	27.5	28	*99.2	26.8	26	

	Pleasantness	AUC	SD	n	AUC	SD	n	
Threshold	<50%	100.4	31.2	9	78.0	32.0	13	**0.023**
	>50%	73.6	30.7	28	89.6	34.5	29	
Preferred	<50%	72.6	30.1	9	77.3	32.2	16	0.809
	>50%	82.6	33.5	28	91.3	34.3	26	

AUC; area under the curve, HC; healthy control, n; participant number; PLWH; people living with HIV; SD; Standard Deviation. P-value; significance level <0.05 (only significant values for interaction effects shown, *^+^ indicate significant pairwise associations). Two-way ANOVA, was used throughout.

### 3.3 Genetics, health variables, salt taste and salt intake

There was an interaction between rs4790522 and health status on BMI (p = 0.005). Specifically, PLWH in the AA genotype group had a lower BMI (21.0 ± 3.9 kg/m^2^) when compared to HC in the AA genotype group (27.9 ± 4.9 kg/m^2^; p = 0.001; Table 5). Additionally, HC in the AA genotype group (27.9 ± 4.9 kg/m^2^) had a higher BMI than those in the AC/CC genotype group (23.6 ± 4.9 kg/m^2^; p = 0.010; Table 5).

**TABLE 5:** BMI, SBP, DBP, and pulse by rs4790522 and rs239345 genotype, within and between PLWH and HC.

TRPV1 rs4790522	PLWH			HC			p-value
	BMI	SD	n	BMI	SD	n	
AA	*21.0	3.9	9	*^+^27.8	4.9	9	**0.005**
AC/CC	23.4	3.5	24	^+^23.6	4.6	33	
SBP							
AA	127.4	22.8	9	116.2	13.4	9	0.264
AC/CC	115.4	17.3	24	114.2	14.3	33	
DBP							
AA	77.2	12.5	9	71.1	11.1	9	0.512
AC/CC	72.1	12.4	24	70.0	9.9	33	
Pulse							
AA	76.7	10.2	9	74.3	12.3	9	0.389
AC/CC	79.5	12.6	24	71.2	13.1	33	

SCNN1B rs239345	BMI						
TT	21.5	3.5	10	*22.0	4.0	11	0.619
AT/AA	23.8	3.5	21	*25.4	4.9	31	
SBP							
TT	113.0	19.5	10	115.0	13.2	11	0.255
AT/AA	122.4	19.5	21	114.5	14.4	31	
DBP							
TT	67.5	7.9	10	68.7	11.8	11	0.188
AT/AA	77.3	13.8	21	70.8	9.6	31	
Pulse							
TT	73.5	11.9	10	73.5	12.0	11	0.109
AT/AA	81.7	11.0	21	71.3	13.3	31	

BMI; body mass index; DBP; diastolic blood pressure; HC; healthy control, n; participant number; PLWH; people living with HIV, SBD; systolic blood pressure, SCNN1B, Sodium Channel Epithelial 1 Subunit (TT, and AT/TT); SD; standard deviation; TRPV1, transient receptor potential cation channel subfamily V member 1 gene (AA, and AC/CC). P-value; significance level <0.05 (only significant values for interaction effects shown, *^+^ indicates significant pairwise associations). Two-way ANOVA, was used throughout.

Bold values are indicated where the p value <0.05.

There was no interaction between rs239345 and health status on BMI (p > 0.05). However pairwise comparisons revealed, HC in the TT genotype group had a lower BMI (21.5 ± 3.5 kg/m^2^) than those in the AT/AA genotype group (23.8 ± 3.5 kg/m^2^; p = 0.025; Table 5).

There was no interaction between rs4790522 or rs239345 and health status on SBP, DBP, or pulse (p > 0.05; Table 5).

As exploratory and hypothesis generating analysis, genotype and salt intake (>/<8 g/d) were explored as influencers of health variables (Supplementary Table S1). There was an interaction between rs4790522 genotype, salt intake and BMI (p = 0.022), PLWH with the AA genotype who consumed less than 8 g/d salt had a lower BMI (18.2 ± 3.4 kg/m^2^) when compared to HC with the AA genotype who consumed less than 8 g/d salt (27.0 ± 5.6 kg/m^2^; p = 0.018) and HC with the AA genotype who consumed more than 8 g/d salt (23.4 ± 3.6 kg/m^2^; p = 0.009). No interaction was found between rs4790522 genotype, salt intake and SBP, DBP, or pulse (p > 0.05).

There was an interaction between rs239345, salt intake, and SBP (p = 0.037) and DBP (p = 0.031). PLWH with the AT/AA genotype who consumed less than 8 g/d salt had a higher SBP (142.0 ± 26.1 mmHg) and DBP (92.0 ± 7.0 mmHg) than almost all other groupings. Data shown in Supplementary Figure S1. No interaction was found between rs4790522 genotype, salt intake and BMI or pulse.

Further, BMI, SBP, and DBP did not differ between cohorts (p > 0.05). PLWH had a higher pulse rate than HC (p = 0.013; Table 6). Pairwise comparisons revealed that PLWH who consumed less than 8 g/d salt had a lower BMI (20.4 ± 3.0 kg/m^2^) than HC who consumed less than 8 g/d salt (24.3 ± 4.3 kg/m^2^; p = 0.009) and HC who consumed more than 8 g/d salt (24.3 ± 4.3 kg/m^2^; p 0.006).

**TABLE 6:** BMI, SBP, DBP, and pulse rate between PLWH and HC, and by median salt intake.

Variable	PLWH			HC			p-value
	Mean	SD	n	Mean	SD	n	
BMI (kg/m^2^)	22.7	3.7	37	24.5	4.9	42	0.142
<8 g/d	*^+^20.4	3.0	24	*24.3	4.3	25	0.107
>8 g/d	24.0	3.4	13	^+^24.9	5.8	17	
SBP (mmHg)	118.9	12.4	37	114.7	10.1	42	0.361
<8 g/d	125.0	23.9	24	114.8	12.7	25	0.289
>8 g/d	115.6	16.6	13	114.4	16.1	17	
DBP (mmHg)	73.3	11.7	37	70.3	10.1	42	0.233
<8 g/d	77.8	13.5	24	71.2	9.5	25	0.381
>8 g/d	70.8	11.3	13	68.9	11.0	17	
Pulse (bpm)	78.9	11.7	37	71.9	12.8	42	**0.013**
<8 g/d	74.2	12.8	24	71.2	14.3	25	>0.050. 332
>8 g/d	81.5	7.7	13	72.8	12.1	17	

BMI; body mass index; DBP; diastolic blood pressure; g/d; grams/day; HC; healthy control, n; participant number; PLWH; people living with HIV, SBD; systolic blood pressure, SD; Standard Deviation. </>8 g/d; grams per day salt. P-value; significance level <0.05 (only significant values for interaction effects shown, */^+^indicate significant pairwise associations). Independent T-test or Two Way ANOVA, was used throughout.

BMI, SBP, DBP, and Pulse did not differ based on salt threshold, and there was no interaction between disease status and threshold on health variables. Disease status and preferred sample influenced SBP and DBP (p = 0.025, 0.046, respectively), however pairwise comparisons were not significant but revealed PLWH with low preferred sample concentration (<50%) had a higher pulse rate (83.8 ± 13.4) than HC with a high preferred sample concentration (>50%; 68.9 ± 14.3; 0.010; Table 7).

**TABLE 7:** BMI, SBP, DBP, and pulse rate between PLWH and HC, and by threshold and preferred sample concentration.

Variable	Threshold	PLWH			HC			p-value
		Mean	SD	n	Mean	SD	n	
BMI (kg/m2)	<50%	21.1	4.4	9	25.1	5.6	13	0.181
	>50%	23.3	3.4	28	24.3	4.6	29	
SBP (mmHg)	<50%	122.4	28.4	9	117.1	15.4	13	0.899
	>50%	117.8	16.5	28	113.6	13.4	29	
DBP (mmHg)	<50%	73.9	14.3	9	73.5	12.2	13	0.497
	>50%	73.1	12.0	28	68.8	8.8	29	
Pulse (bpm)	<50%	80.8	14.8	9	70.9	11.5	13	0.540
	>50%	78.3	10.8	28	72.3	13.6	29	
Preferred								
BMI (kg/m2)	<50%	23.2	4.4	9	25.1	5.2	16	0.892
	>50%	22.6	3.5	28	24.2	4.8	26	
SBP (mmHg)	<50%	110.2	11.1	9	119.2	15.1	16	**0.025**
	>50%	121.7	21.1	28	111.9	12.7	26	
DBP (mmHg)	<50%	70.4	13.2	9	74.8	11.1	16	**0.046**
	>50%	74.2	12.2	28	67.5	8.5	26	
Pulse (bpm)	<50%	*83.8	13.4	9	76.7	8.3	16	0.828
	>50%	77.3	10.9	28	*68.9	14.3	26	

BMI; body mass index; DBP; diastolic blood pressure; g/d; grams/day; HC; healthy control, n; participant number; PLWH; people living with HIV, SBD; systolic blood pressure, SD; standard deviation; </>50%; less or more than the median. P-value; significance level <0.05 (only significant values for interaction effects shown, * indicates significant pairwise associations). Two-way ANOVA, was used throughout.

Bold values are indicated where the p value <0.05.

No interaction was found between rs4790522 or rs239345 and health status on salt intake (g/d, p > 0.05; Supplementary Table S2).

**FIGURE 4:**
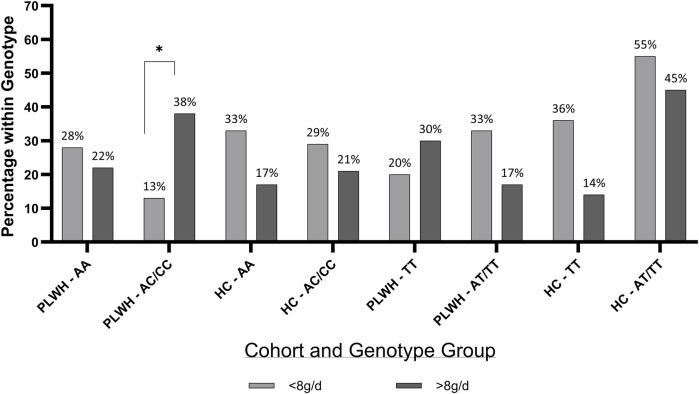
Percentage of PLWH and HC in *TRPV1* rs4790522 and *SCNN1B* rs239345 within genotype groups by salt intake. g/d; grams/day; HC; Healthy Control, PLWH; people living with HIV, SCNN1B, Sodium Channel Epithelial 1 Subunit (TT and AT/TT); TRPV1, transient receptor potential cation channel subfamily V member 1 gene (AA and AC/CC). P-value; significance level <0.05. Chi Squared test was used throughout.

When the distribution of participants within salt intake groups (>/<8 g/d) was assessed, more PLWH (40%) with the AC/CC genotype consumed >8 g salt/day, when compared to those who consumed <8 g salt/day (10%; Figure 4). No significance was found in HC, and salt intake did not differ based on rs239345 genotype in PLWH nor HC.

More PLWH and HC with a high (>50% concentration) threshold had the rs4790522 AC/CC genotype (61%, p < 0.001; 70%, p = 0.014, respectively; Supplementary Figure S2A), however this was also true for the HC AA genotype group (18%, p = 0.014). More PLWH and HC with a high (>50%) preferred sample concentration had the rs4790522 AC/CC genotype (51%, p < 0.001; 48%, p = 0.024, respectively), and the *SCNN1B* rs239345 genotype (45%, p = 0.026; 43%, p = 0.026, respectively; Supplementary Figure S2B).

Neither AUC for intensity or pleasantness differed based on rs4790522 nor rs239345 in PLWH and HC (p > 0.05; Supplementary Table S3).

### 3.4 Discussion

This study investigated the differences between salt intake and salt taste perception and the interplay between genetic variations in salt taste receptors (SCNN1B rs239345 and TRPV1 rs4790522) with salt taste perception, dietary salt intake, and cardiovascular health parameters in Zambian adults, with a focus on PLWH. The key findings reveal that PLWH consumed significantly more salt than HC (9.01 vs. 7.11 g/day) and exhibited reduced salt intensity perception (AUC: 94.8 vs. 109.2). Genetic analyses highlighted that PLWH carrying the TRPV1 rs4790522 AC/CC genotype had higher salt intake (9.4 vs. 7.3 g/day) than HC, and the TRPV1 rs4790522 SNP was associated with BMI, suggesting a gene-environment-disease interaction, suggesting a potential gene-environment-disease interaction. Our results present novel findings in an understudied cohort and thus warrant further investigation.

#### 3.4.1 Elevated salt intake and impaired salt perception in PLWH

The observed higher salt intake in PLWH aligns with prior literature related to taste dysfunction (Fasunla et al., 2016; Prakash et al., 2024). Reduced intensity perception may drive compensatory salt use, exacerbating hypertension risk, a critical concern given the high CVD burden in SSA (Minja et al., 2022). However, a direct correlation between salt intake and taste thresholds was not found in this study. Literature in this area is heterogenous with some reporting an association (Sari et al., 2022) whilst others, alike this study, reporting no association (Tan et al., 2021). This suggests multifactorial influences, including cultural dietary practices or socioeconomic factors not captured here. Additionally, a higher salt intake in PLWH may be due to HIV-associated neuropathies, recurrent infections, ART effects, or the increased age of the PLWH cohort compared to the HC (Fasunla et al., 2017; Barragán et al., 2018; Masenga et al., 2019). Taste perception has been reported to decline with age, across a range of concentrations (Barragán et al., 2018) regarding salt, whether this is associated with dietary intake and aging is disputed (Tepper, 2008; Liem and Russell, 2019). Some have demonstrated that aging is associated with a declined taste sensitivity and, subsequently, diet quality (Jeon et al., 2021). However, this literature generally refers to adults over 65 years and therefore cannot be directly compared to the current findings. Therefore, further research on larger aged-balanced cohorts is required to comprehensively determine the drivers of salt intake in PLWH.

Moreover, although taste has been stated as indicative of dietary intake (Tan et al., 2021), many aspects of taste contribute to flavour (IQWiG, 2023). This study aimed to assess salt taste intensity and pleasantness as a driver of salt intake, which has been demonstrated in previous literature (Pilic et al., 2020), however flavour is a complex interplay and further research should consider assessment of all the six researched tastes (Besnard et al., 2016; IQWiG, 2023) and examine all aspects of taste perception, including identification, threshold, and discrimination testing, alongside taste liking (Technical Committee, 2011; Liem and Russell, 2019).

#### 3.4.2 Genetic susceptibility and salt intake and salt perception

The TRPV1 rs4790522 AC/CC genotype demonstrated a higher frequency in those with a higher salt intake in PLWH, representing a novel finding. Similarly, Chamoun et al. (2018) reported that the C allele was associated with a significantly higher salt preference in preschool children of the Guelph region, Canada, compared to the A allele. In keeping, Tapanee et al. (2021) found that adults with hypertension carrying the AA genotype exhibited a higher salt recognition threshold, which as discussed previously, may be indicative of a higher dietary intake. However, this current study found no association with rs4790522 salt intensity or pleasantness. Given the variation in age and ethnicity across these cohorts, direct comparison is unsuitable. Further investigation of rs4790522 in relation to salt intake, preference, and perception is warranted within more demographically and clinically homogeneous population, particularly because the minor allele (A) frequency (MAF) is approximately 10% higher in African cohorts than in Caucasians (Auton et al., 2015), therefore if negative dietary implications are apparent then importance of this research is heightened.

No associations between SCNN1B rs239345 and salt intake or perception were identified in this study. This contrasts with findings from Caucasian cohorts; for example, Barragán et al. (2018) reported that the AA genotype was associated with heightened salt intensity perception in Europeans, whereas Dias et al. (2013) found that, in a Canadian cohort, AA carriers perceived salt less intensely than those with the T allele. This inconsistency highlights the need for further population-specific genetic research, particularly because the MAF is also higher in African cohorts (Auton et al., 2015). Overall, such research has the potential to contribute to personalized nutrition (PN) practices. PN is a leading method for dietary change and improved health (Hinojosa-Nogueira et al., 2024), however, related research is predominantly from westernised, affluent societies (Otterbring and Folwarczny, 2024). PN, incorporating genetics, runs the risk of remaining an inaccessible, expensive method of prevention and treatment (Mathers, 2019). Therefore, exploring phenotypes that are predictive of genetic predispositions offers a more accessible approach. For example, prediction of genetic predisposition has been previously demonstrated regarding bitter taste ability (Aoki et al., 2023), but to date, not in other taste modalities.

#### 3.4.3 Health markers of hypertension risk

Notably, PLWH carrying the rs4790522 AA genotype exhibited lower BMIs than HC (21.0 vs. 27.9 kg/m^2^, p = 0.001), this finding is supportive that a higher perceived intensity may result in a lower dietary consumption of salt (Hayes et al., 2010). However, this finding is not consistent with recent literature, for example, systematic review by Tan et al. (2021) that reports salt sensitivity and intensity did not relate to dietary intake but dietary intake was better predicted by salt taste hedonic ratings. Additionally, a lower BMI in PLWH may reflect HIV-related metabolic dysregulation or ART effects (Masenga et al., 2019). Without research carried out in larger cohorts, powered for genetic and BMI groupings these findings should be considered hypothesis generating only.

Lastly, while overall BP did not differ between groups, PLWH with the SCNN1B rs239345 AT/AA genotype and high salt intake had elevated SBP/DBP (142.0/92.0 mmHg), suggesting a genetic predisposition to salt-sensitive hypertension. This aligns with evidence that ENaC variants modulate renal sodium handling and BP (Tapanee et al., 2021). The higher pulse rate in PLWH (78.9 vs. 71.9 bpm, p = 0.013) further signals cardiovascular strain, warranting longitudinal monitoring.

#### 3.4.4 Limitations and strengths

This study presents novel findings in an under-researched population; however, it is not without limitations. Most notably, this study is limited by sample size (n = 79), reducing statistical power for genetic associations. Additionally, the age disparity between PLWH (older) and HC may confound outcomes related to differences in taste, due to taste sensitivity often declining with age (Barragán et al., 2018). This does not deter from any genetic related conclusions, however, results reported must be considered hypothesis-generating only, supporting future research in sub-Saharan African populations, particularly among PLHW and/or hypertension. Further, dietary intake was self-reported, which is inherently prone to error (National Research Council, 1986; Amoutzopoulos et al., 2020). Although a 5-step multiple pass dietary recall method was used, with reported accuracy within 10% of actual intake (Conway et al., 2003), day-to-day variability and systematic bias remain potential sources of error (National Research Council, 1986). Finally, cross-sectional design limits causal inferences, thus, the long-term implications of taste perception and salt intake require further investigation. Despite these limitations, the novel integration of genetic, sensory, and clinical data in an understudied population represents a substantial strength and provides a foundation for targeted, population-specific interventions.

#### 3.4.5 Public health relevance

In a region grappling with dual burdens of HIV and CVD, addressing modifiable risk factors like salt intake through genetic and sensory profiling could mitigate morbidity. Policy initiatives promoting low-salt diets and routine taste assessments in PLWH clinics may enhance health outcomes in this vulnerable population.

### 3.5 Conclusion

Overall, this work demonstrates that within this cohort, PLWH consume more salt than HC, which may be driven by a reduced salt perception. Differences were found in the association between the TRPV1 rs4790522 SNP, salt intake and BMI profiles, between PLWH and HC, suggesting a gene-environment-disease interaction. These findings highlight the need for further research in SSA, which should employ larger cohorts, longitudinal designs, and objective sodium biomarkers (e.g., 24-h urine) to clarify causal pathways. Such design would allow a wider assessment of polygenic influencers of salt taste perception and cardiovascular health. Further, when investigating PLWH, ART-specific effects on taste and metabolism should be explored. Taken together, such research could influence salt-reduction strategies tailored to PLWH, particularly those with genetic risk variants.

## Data Availability

The raw data supporting the conclusions of this article will be made available by the authors, without undue reservation.
