# Supplementary material for: Salt taste perception, dietary salt intake, cardiovascular health and genetic variation in Zambian adults with HIV
Source: Front Physiol. 2025 Oct 14;16:1616785. doi: 10.3389/fphys.2025.1616785 (PMC12558775; doi:10.3389/fphys.2025.1616785)
Supplement: Supplementary file 1 [file Table1.docx]

***Supplementary Table 1.*** *Salt intake (>/< 8g/d) and rs4790522 and rs239345 genotype by BMI, SBP, DBP, and pulse by, within and between PLWH and HC.*

| TRPV1 rs4790522 | | PLWH | | | HC | | |  |
| --- | --- | --- | --- | --- | --- | --- | --- | --- |
| Salt |  | BMI (kg/m^2^) | SD | n | Mean | SC | n | p-value |
| <8g/d | AA | *^+^18.2 | 3.4 | 5 | *27.0 | 5.6 | 6 | **0.028** |
|  | AC/CC | 21.7 | 3.6 | 6 | 23.4 | 3.6 | 19 |  |
| >8g/d | AA | ^+^24.4 | 3.6 | 4 | 29.5 | 3.1 | 3 |  |
|  | AC/CC | 23.9 | 3.4 | 18 | 23.9 | 5.8 | 14 |  |
|  |  | SBP (mmHg) |  |  |  |  |  |  |
| <8g/d | AA | 134.8 | 27.0 | 5 | 123.0 | 10.0 | 6 | 0.511 |
|  | AC/CC | 114.0 | 22.4 | 6 | 112.3 | 12.6 | 19 |  |
| >8g/d | AA | 118.3 | 17.0 | 4 | 102.7 | 7.5 | 3 |  |
|  | AC/CC | 115.9 | 16.0 | 18 | 116.9 | 16.4 | 14 |  |
|  |  | DBP (mmHg) |  |  |  |  |  |  |
| <8g/d | AA | 80.2 | 14.6 | 5 | 75.5 | 10.0 | 6 | 0.759 |
|  | AC/CC | 73.8 | 15.9 | 6 | 69.8 | 9.2 | 19 |  |
| >8g/d | AA | 73.5 | 9.5 | 4 | 62.3 | 8.5 | 3 |  |
|  | AC/CC | 71.6 | 11.5 | 18 | 70.4 | 11.2 | 14 |  |
|  |  | Pulse (bpm) | |  |  |  |  |  |
| <8g/d | AA | 74.2 | 7.3 | 5 | 75.0 | 11.0 | 6 | 0.812 |
|  | AC/CC | 78.0 | 9.5 | 6 | 70.1 | 12.4 | 19 |  |
| >8g/d | AA | 79.8 | 11.0 | 4 | 73.0 | 17.3 | 3 |  |
|  | AC/CC | 80.0 | 13.7 | 18 | 72.8 | 14.3 | 14 |  |
| SCNN1B rs239345 | |  |  |  |  |  |  |  |
|  |  | BMI (kg/m^2^) |  |  |  |  |  |  |
| <8g/d | TT | 21.7 | 3.5 | 5 | *22.1 | 2.8 | 8 | 0.505 |
|  | AT/AA | ^*+X^20.8 | 3.8 | 5 | ^+^25.3 | 4.6 | 17 |  |
| >8g/d | TT | 21.3 | 3.6 | 5 | 21.7 | 7.4 | 3 |  |
|  | AT/AA | ^X^24.7 | 3.0 | 16 | 25.6 | 5.5 | 14 |  |
|  |  | SBP (mmHg) |  |  |  |  |  |  |
| <8g/d | TT | 112.2 | 17.3 | 5 | *112.1 | 8.2 | 8 | **0.045** |
|  | AT/AA | ^*+XY^142.0 | 26.1 | 5 | ^X^116.1 | 14.4 | 17 |  |
| >8g/d | TT | 113.8 | 22.2 | 5 | 122.7 | 22.5 | 3 |  |
|  | AT/AA | ^+^116.3 | 12.6 | 16 | ^Y^112.6 | 14.8 | 14 |  |
|  |  | DBP (mmHg) |  |  |  |  |  |  |
| <8g/d | TT | *67.0 | 8.0 | 5 | ^+^67.9 | 6.5 | 8 | **0.029** |
|  | AT/AA | ^*+XYWZ^92.0 | 7.0 | 5 | ^X^72.7 | 10.4 | 17 |  |
| >8g/d | TT | ^Y^68.0 | 8.1 | 5 | 71.0 | 23.1 | 3 |  |
|  | AT/AA | ^W^72.8 | 12.0 | 16 | ^Z^68.5 | 8.2 | 14 |  |
|  |  | Pulse (bpm) | |  |  |  |  |  |
| <8g/d | TT | 75.2 | 7.3 | 5 | 71.0 | 9.8 | 8 | 0.273 |
|  | AT/AA | 79.0 | 9.7 | 5 | 71.4 | 13.3 | 17 |  |
| >8g/d | TT | 71.8 | 15.5 | 5 | 80.3 | 16.9 | 3 |  |
|  | AT/AA | 82.6 | 11.6 | 16 | 71.2 | 13.8 | 14 |  |

*BMI; body mass index, DBP; diastolic blood pressure, g/d; grams/day of salt; HC; Healthy Control, PLWH; people living with HIV, SBP; systolic blood pressure, SCNN1B, Sodium Channel Epithelial 1 Subunit (TT and AT/TT); TRPV1, transient receptor potential cation channel subfamily V member 1 gene (AA and AC/CC). P-value; significance level <0.05. Significant values are shown in figure. Two-way ANOVA was used throughout.*
