# Supplementary figures and images for: Salt taste perception, dietary salt intake, cardiovascular health and genetic variation in Zambian adults with HIV
Source: Front Physiol. 2025 Oct 14;16:1616785. doi: 10.3389/fphys.2025.1616785 (PMC12558775; doi:10.3389/fphys.2025.1616785)

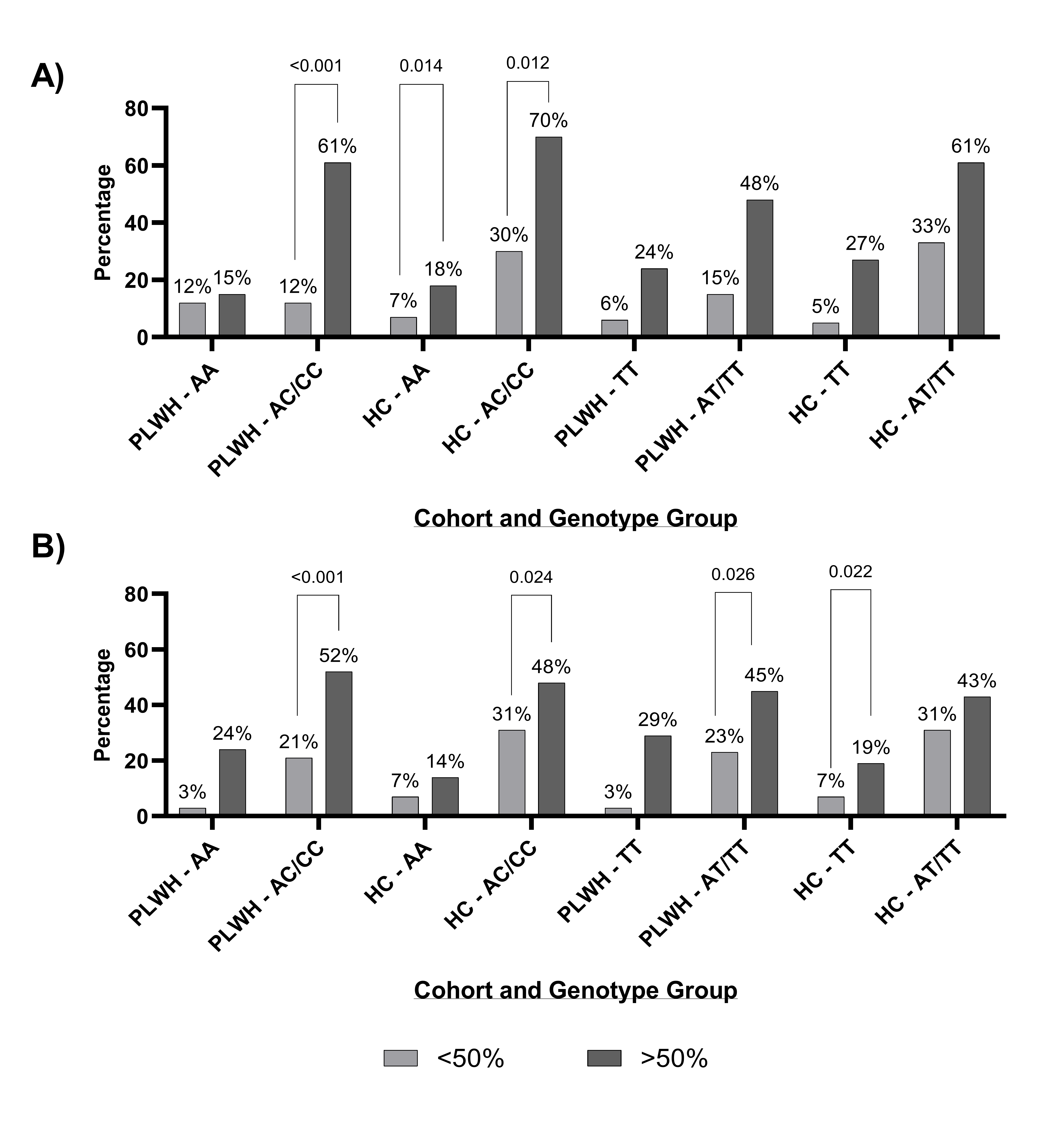

Supplement: Supplementary file 2 [file Image2.tif]
