# Supplementary material for: Salt taste perception, dietary salt intake, cardiovascular health and genetic variation in Zambian adults with HIV
Source: Front Physiol. 2025 Oct 14;16:1616785. doi: 10.3389/fphys.2025.1616785 (PMC12558775; doi:10.3389/fphys.2025.1616785)
Supplement: Supplementary file 4 [file Table2.docx]

**Supplementary Table 2.** Salt intake by *TRPV1* rs4790522 and *SCNN1B* rs239345 in PLWH and HC

|  | PLWH | | | HC | | |  |
| --- | --- | --- | --- | --- | --- | --- | --- |
|  | **Salt (g/d)** | **SD** | **n** | **Salt (g/d)** | **SD** | **n** | **p-value** |
| TRPV1 rs4790522 | |  |  |  |  |  |  |
| AA | 7.3 | 2.1 | 9 | 6.8 | 2.3 | 9 | 0.647 |
| AC/CC | 9.4 | 4.1 | 24 | 8.0 | 3.8 | 33 |  |
| SCNN1B rs239345 | |  |  |  |  |  |  |
| TT | 8.3 | 2.7 | 10 | 6.3 | 1.9 | 11 | 0.563 |
| AT/AA | 9.1 | 4.2 | 21 | 8.2 | 3.9 | 31 |  |

*HC; Healthy Control, PLWH; people living with HIV, SCNN1B, Sodium Channel Epithelial 1 Subunit (TT and AT/TT); SD; standard deviation, TRPV1, transient receptor potential cation channel subfamily V member 1 gene (AA and AC/CC). P-value; significance level <0.05. Chi Squared test was used throughout.*
