# Supplementary material for: Salt taste perception, dietary salt intake, cardiovascular health and genetic variation in Zambian adults with HIV
Source: Front Physiol. 2025 Oct 14;16:1616785. doi: 10.3389/fphys.2025.1616785 (PMC12558775; doi:10.3389/fphys.2025.1616785)
Supplement: Supplementary file 5 [file Table3.docx]

**Supplementary** **Table 3**. AUC for intensity and pleasantness of salt by rs4790522 and rs239345 genotype, within and between PLWH and HC.

|  |  |  | | PLWH | | | HC | | |  |
| --- | --- | --- | --- | --- | --- | --- | --- | --- | --- | --- |
|  | **AUC** |  | | **Mean** | **SD** | **N** | **Mean** | **SD** | **N** | **p-value** |
| TRPV1 rs4790522 |  | | **Intensity** | | | | | | |  |
| AA |  |  | | 93.8 | 17.0 | 9 | 114.6 | 29.0 | 9 | 0.467 |
| AC/CC |  |  | | 94.5 | 33.3 | 24 | 104.8 | 26.3 | 33 |  |
|  |  | | **Pleasantness** | | | | | | |  |
| AA |  |  | | 98.0 | 29.5 | 10 | 105.8 | 26.5 | 11 | 0.956 |
| AC/CC |  |  | | 96.5 | 28.1 | 21 | 103.5 | 27.1 | 31 |  |
| SCCN1B rs239345 |  | | **Intensity** | | | | | | |  |
| TT |  |  | | 98.5 | 30.5 | 9 | 80.2 | 41.3 | 9 | 0.126 |
| AT/AA |  |  | | 73.7 | 32.4 | 24 | 83.3 | 32.1 | 33 |  |
|  |  | | **Pleasantness** | | | | | | |  |
| TT |  |  | | 66.2 | 24.4 | 10 | 93.4 | 33.7 | 11 | 0.074 |
| AT/AA |  |  | | 86.7 | 36.8 | 21 | 82.0 | 34.1 | 31 |  |

*HC; Healthy Control, PLWH; people living with HIV, SCNN1B, Sodium Channel Epithelial 1 Subunit (TT and AT/TT); SD; standard deviation, TRPV1, transient receptor potential cation channel subfamily V member 1 gene (AA and AC/CC). P-value; significance level <0.05. Two-way ANOVA was used throughout.*
